# Supplementary material for: Rapid Whole-Body FDG PET/MRI in Oncology Patients: Utility of Combining Bayesian Penalised Likelihood PET Reconstruction and Abbreviated MRI
Source: Diagnostics (Basel). 2023 May 26;13(11):1871. doi: 10.3390/diagnostics13111871 (PMC10252718; doi:10.3390/diagnostics13111871)
Supplement: Supplementary file 1 [file diagnostics-13-01871-s001.zip › diagnostics-2348279-supplementary.pdf]

diagnostics

Article

Supplementary Materials: Rapid Whole-body FDG PET/MRI in Oncology Patients: Utility of Combining Bayesian Penalized Likelihood PET Reconstruction and Abbreviated MRI

Junko Inukai-Inoue[1], Munenobu Nogami[2,3,*], Miho Tachibana[1], Feibi Zeng[2], Tatsuya Nishitani[2], Kazuhiro Kubo[2], and Takamichi Murakami[1*]

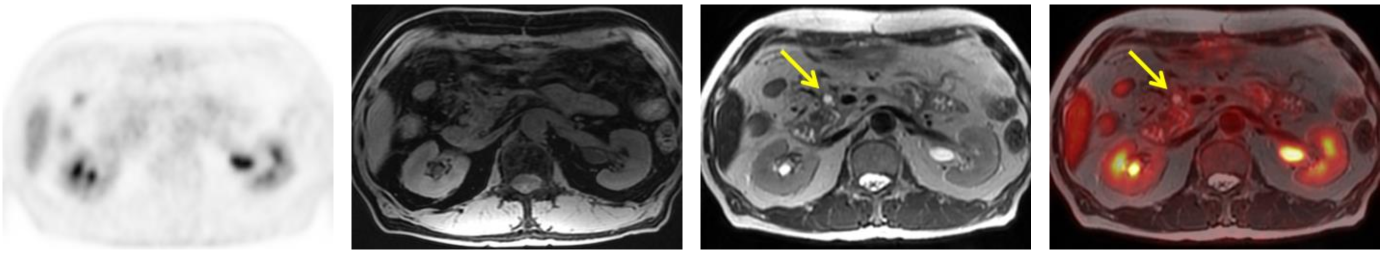

(**a**) (**b**) (**c**) (d)

Figure S1. A case of pancreatic cystic lesion without significant FDG uptake. The lesion was difficult to detect on abb-MRI but can clearly be seen on std-MRI, including T2WI. (a) $OSEM_{2.5}$, (b) Fat suppression T1WI, (c) T2WI, (d) $OSEM_{2.5}$/std-MRI.
